# struct: an R/Bioconductor-based framework for standardized metabolomics data analysis and beyond

**DOI:** 10.1093/bioinformatics/btaa1031

**Published:** 2020-12-27

**Authors:** Gavin Rhys Lloyd, Andris Jankevics, Ralf J M Weber

**Affiliations:** Phenome Centre Birmingham and School of Biosciences, University of Birmingham, Birmingham, B15 2TT, UK

## Abstract

**Summary:**

Implementing and combining methods from a diverse range of R/Bioconductor packages into ‘omics’ data analysis workflows represents a significant challenge in terms of standardization, readability and reproducibility. Here, we present an R/Bioconductor package, named struct (Statistics in R using Class-based Templates), which defines a suite of class-based templates that allows users to develop and implement highly standardized and readable statistical analysis workflows. Struct integrates with the STATistics Ontology to ensure consistent reporting and maximizes semantic interoperability. We also present a toolbox, named structToolbox, which includes an extensive set of commonly used data analysis methods that have been implemented using struct. This toolbox can be used to build data-analysis workflows for metabolomics and other omics technologies.

**Availability and implementation:**

struct and structToolbox are implemented in R, and are freely available from Bioconductor (http://bioconductor.org/packages/struct and http://bioconductor.org/packages/structToolbox), including documentation and vignettes. Source code is available and maintained at https://github.com/computational-metabolomics.

## 1 Introduction

The development of computational workflows to analyse complex multivariate biological datasets has become commonplace across an ever growing number of scientific disciplines. The R language is often used to develop such workflows, and there is a large number of publicly available packages that enhance its functionality. CRAN and Bioconductor are two examples of free, open source repositories for R packages. Bioconductor has a strong focus on molecular biology, while CRAN is more generic. Implementing and combining functionalities from a diverse range of R/Bioconductor packages into robust, readable and reproducible scripts and workflows can be challenging and time consuming. The lack of reproducibility and reusability of such scripts and workflows is increasingly being scrutinized by the ‘omics’ user communities, scientific journals and funding bodies, highlighting the need for a standardized framework to address these limitations. This led us to develop struct (Statistics in R using Class-based Templates), which includes a suite of S4 class-based templates (i.e. model, sequence, iterator, chart and metric classes) to facilitate the standardization of R-based workflows for statistics and machine learning. S4 is an R-based system for object oriented programming and is commonly used in Bioconductor. Struct integrates with the STATistics Ontology (STATO) to ensure consistent reporting and provide interoperability via semantic types (Rocca-Serra et al., 2019). A complementary R/Bioconductor package, named structToolbox, is also presented to demonstrate the implementation and application of struct. The toolbox contains an extensive set of commonly used methods that have been implemented using struct, and which can be used to build preprocessing and data analysis workflows for metabolomics and other omics technologies.

## 2 Materials and methods

Here, we describe each of the main S4 class-based templates included in struct. The templates have been designed to support different functionalities that are required to build a typical statistics and/or machine learning workflow. A set of helper functions (e.g. set_struct_obj and set_obj_method) are available to allow the user to create and develop new objects from one of the pre-defined templates. In this way, users will be able to include data analysis methods beyond those from metabolomics and easily incorporate new analyses into their workflows.

### 2.1 DatasetExperiment class: a data container

**Fig. 1.**
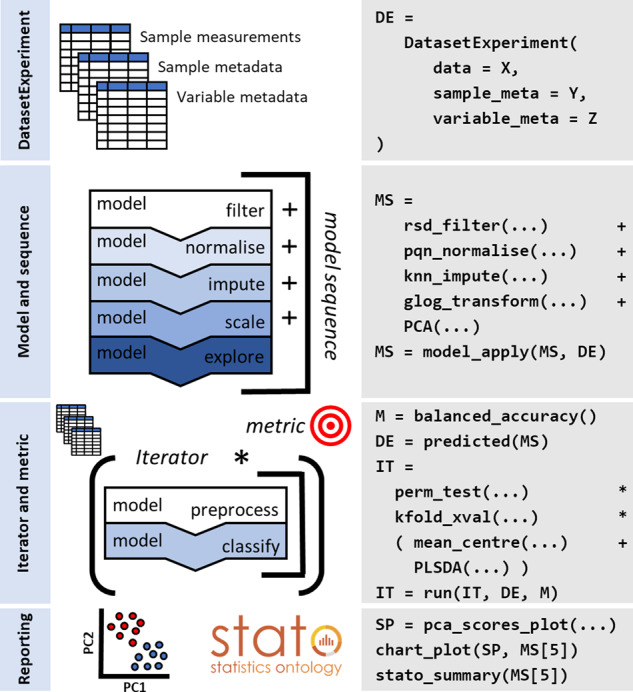
Illustration summarizing the main struct S4 class-based templates and associated pseudo code defining a metabolomics-based workflow for preprocessing and multivariate statistics

The DatasetExperiment class (Fig. 1, defined as DE) is an extension of Bioconductor's SummarizedExperiment class (Morgan *et al.*, 2020). It is used to standardize the format of data matrices and meta data commonly generated by omics studies, and used within a struct-based workflow. The DatasetExperiment object contains three components, sample measurements, sample and variable metadata. They are coordinated in such a way that the samples and/or variables can be subset and/or filtered while remaining the samples and variables in sync.

### 2.2 Model and sequence classes

Struct allows a user to compartmentalize a data analysis workflow into a set of objects derived from the ‘model’ class. More specifically, the ‘model’ class can be used as a template to define and implement statistical or machine learning methods from existing R/Bioconductor packages [e.g. partial least squares-discriminant analysis (PLSDA) and support vector machines (SVM)] and/or bespoke R-code. It can also be used to implement preprocessing steps, such as normalization and scaling. The different class objects can be linked together into a sequence object using the plus (i.e. ‘+’) symbol (Fig. 1, defined as MS). This particular ‘grammar’ is comparable to that employed by TidyVerse (Wickham *et al.*, 2019). A sequence can be executed using the (i) ‘model_train’ method to train each step of the sequence using a training dataset; (ii), the ‘model_predict’ method to apply a trained sequence on a test dataset (i.e. model validation); (iii) ‘model_apply’ to train and predict using the same dataset (sometimes called autoprediction), which is useful for non-supervised and univariate statistical methods (Fig. 1). The outputs of each step in the workflow (i.e. sequence) are retained and accessible by its index number (see Fig. 1, for example MS[5]). This allows the user to explore different steps of the analysis and also allows sequences to be nested within other objects for the purposes of, for example, cross-validation (see Section 2.3). The method ‘predicted’ (Fig. 1) can be used to extract the modified data matrix and metadata, in the form of a DatasetExperiment object, from any step in a sequence.

### 2.3 Iterator and metric classes for model validation

Supervised multivariate analysis requires training and testing phases to validate a model. The struct ‘iterator’ and ‘metric’ classes have been designed to support such approaches. Iterators allow the collection of model outputs over a number of iterations. The asterisk (i.e. ‘*’) symbol can be used to nest a model (or model sequence) inside an iterator, which indicates that the nested model (or sequence) will be used multiple times. As well as model outputs it is useful to accumulate metrics, such as balanced accuracy (Fig. 1, defined as M), over a number of iterations. Both iterator and metric classes can be extended like the other templates (Fig. 1, defined as perm_test and kfold_xval). The ‘run’ method is provided to execute an iterator with a nested model and calculate the input metric for all iterations.

### 2.4 Chart and STATO classes for reporting

Struct includes a ‘chart’ class, which allows specific types of plots to be defined depending on the type of the input object. For DatasetExperiment objects this might be boxplots of a variable/feature in different sample groups, while for models and iterators will be specific to the interpretation of that method, e.g. a scores plot for PLSDA. The plots available for a class can be standardized, e.g. using ggplot themes allowing for consistency in reporting (Wickham et al., 2009). New charts can be added by extending the provided chart class template. All templates can be combined with the STATO template, which enables methods for extracting formal definitions of statistical methods from a snapshot of the STATO repository (Rocca-Serra *et al.*, 2019).

## 3 Implementation and application

To demonstrate the implementation and applicability of struct we have developed a package, named structToolbox, which includes a diverse range of methods commonly used to study metabolomics datasets (Stanstrup et al., 2019). Various preprocessing steps such as filters, normalization and scaling are included as well as univariate statistics (e.g. *t*-test, ANOVA and the non-parametric equivalents), multivariate statistics (e.g. PCA, PLSDA), model validation methods (cross-validation, permutation testing) and machine learning methods (SVM). Comprehensive vignettes are available that demonstrate the application of the structToolbox to a range of public Liquid Chromatography Mass Spectrometry (LCMS), Direct Infusion Mass Spectrometry (DIMS) and Nuclear Magnetic Resonance (NMR)_metabolomics datasets.
